# The effect of transcranial direct current stimulation on bilateral asymmetry and joint angles of the lower limb for females when crossing obstacles

**DOI:** 10.1186/s13102-023-00793-2

**Published:** 2023-12-21

**Authors:** I-Lin Wang, Chin-Yi Gu, Tze-Huan Lei, Che-Hsiu Chen, Chih-Hui Chiu, Yu Su

**Affiliations:** 1 College of Physical Education, Hubei Normal University, 435002 Huangshi, Hubei China; 2 Department of Sport Performance, National Taiwan University of Sport, 404 Taichung, Taiwan; 3 Department of Exercise Health Science, National Taiwan University of Sport, 404 Taichung, Taiwan; 4 Graduate Institute, Jilin Sport University, 130022 Changchun, China

**Keywords:** tDCS, Gait asymmetry, Swing time, Stance time, Lower extremity kinematics

## Abstract

**Background:**

Gait asymmetry is often accompanied by the bilateral asymmetry of the lower limbs. The transcranial direct current stimulation (tDCS) technique is widely used in different populations and scenarios as a potential tool to improve lower limb postural control. However, whether cerebral cortex bilateral tDCS has an interventional effect on postural control as well as bilateral symmetry when crossing obstacles in healthy female remains unknown.

**Methods:**

Twenty healthy females were recruited in this prospective study. Each participant walked and crossed a height-adjustable obstacle. Two-way repeated ANOVA was used to evaluate the effect of group (tDCS and sham-tDCS) and height (30%, 20%, and 10% leg length) on the spatiotemporal and maximum joint angle parameters for lower limb crossing obstacles. The Bonferroni post-hoc test and paired t-test were used to determine the significance of the interaction effect or main effect. The statistically significant differences were set at p < 0.05.

**Results:**

The Swing time (SW) gait asymmetry (GA), Stance time (ST) GA, leading limb hip-knee-ankle maximum joint angles and trailing limb hip-knee maximum joint angles decreased in the tDCS condition compared to the sham-tDCS condition at 30%, 20% leg’s length crossing height except for 10% leg’s length, whereas there was a significant decrease in SW/ST GA between the tDCS condition and the sham-tDCS condition at 30%, 20%, 10% leg’s length crossing height (P < 0.05).

**Conclusion:**

We conclude that tDCS intervention is effective to reduce bilateral asymmetry in spatio-temporal parameters and enhance dynamic balance in female participants during obstacle crossing when the heights of the obstacles were above 10% of the leg’s length.

**Trial registration No:**

ChiCTR2100053942 (date of registration on December 04, 2021). Prospectively registered in the Chinese Clinical Trial Registry.

**Supplementary Information:**

The online version contains supplementary material available at 10.1186/s13102-023-00793-2.

## Introduction

Neurostimulation is widely used as a technique to alter neural activity currently, and its safety also broadens the possibility of conducting validation studies with different populations in different fields [1], among which the bipolar-specific non-invasive technique - transcranial direct current stimulation (tDCS) - has been found in previous studies to have a potentially beneficial effect on alleviating central fatigue, improving neuromuscular function and endurance sports performance [2–4]. Based on M1 as primary motor area can encode motor execution [5],tDCS applied bilaterally to the M1 motor cortex has been shown to increase knee flexor and extensor strength in healthy males on the non-dominant side [6]. In addition, Hou et al. [6] showed that anodal tDCS can instantly improve the static and dynamic balance ability of the healthy young people standing on one leg as well as jumping over a 10 cm obstacle [7]. This potentially indicates that the use of tDCS may potentially enhance dynamic balance ability in daily living setting environment involving with multiple crossing and jumping abilities. However, no previous studies are available to examine whether tDCS can enhance dynamic balance ability from a lower level of balance task to a higher level of balance task in female population. This is important given the fact that current results on tDCS are mainly generated from male and limited studies are available to examine this effect in female population. Therefore, an investigation into this issue would probably reduce the falling risk in female population and thereby reducing their risk of musculoskeletal injuries during daily living action.

Crossing obstacles is the best method to assess dynamic balance in a daily living action and it is also a very useful tool to examine dynamic balance ability from a lower level of difficulties to a higher level of difficulties. This is due to the fact that crossing obstacle requires the body to make dynamic adjustments to adapt to various heights. The risk of falling may occur when the coordination between the supporting and swinging limbs was reduced [8]. Previous research has shown that tDCS was involved in improving limb postural control ability during dynamic balance tasks [9]. At once, tDCS has a ‘brain doping’ effect that can improve jumping power and limb coordination in skiers on unstable planes [10]. Thus, tDCS may enhance both cognitive and neuromuscular function which subsequently enhances postural control and stability of the lower limbs when crossing the obstacle with increasing the level of difficulties. Moreover, when the lower limbs alternate in the task of propelling the limb forward and supporting the weight shift during walking, the dominant and non-dominant limbs contribute differently [11, 12]. Previous research has suggested that unilateral limb dominance caused by differences in the dominant functions upon the two hemispheres of the brain is one of the causes of bilateral asymmetry in normal gait [11], gait asymmetry associated with natural functional differences between the two limbs can also lead to bilateral musculoskeletal mass and strength imbalances [13]. Simultaneously, greater lower limb asymmetry necessitates more energy to walk, and prolonged asymmetrical walking can place additional strain on the lower limb joints and accelerate degenerative joint changes [14]. Therefore, gait asymmetry is an important factor affecting walking efficiency and safety, reduced lower limb asymmetry allows the limb to make faster transitions in response to disturbances, lowering the risk of falling.

As biomechanical parameters can further analyze the intuitive gait impressions provided by spatiotemporal parameters to understand the causes of asymmetrical gait, also taking into account the existence of sex differences, it has been shown in the past that hormonal fluctuations (e.g. estrogen, progesterone, etc.) during the normal menstrual cycle in healthy women do not affect the ankle dynamic postural control, neuromuscular and biomechanical characteristics [15, 16], therefore the aim of this study was to investigate the effects of cerebral cortex bilateral tDCS interventions and crossing height on the symmetry of spatiotemporal parameters of the lower limb and the maximum joint angle of the leading limb and trailing limb when crossing an obstacle in healthy females. Fatigue induced by various factors during exercise is associated with suboptimal firing rates of relevant motor neurons [1], tDCS intervention has been shown to reduce nueromuscular fatigue and given its effectiveness in enhancing movement control [4], we therefore hypothesized that as the height of the obstacle increased, the tDCS intervention would reduce bilateral asymmetry and the maximum joint angle of the leading or trailing limb when crossing the obstacles.

## Materials and methods

### Participants

An a priori power analysis (G*Power version 3.1.9.4; Heinrich Heine University Düsseldorf, Düsseldorf, Germany) showed that a minimum of 19 participants was required on the basis of conventional α (0.05) and β (0.80) values, and an effect size of 1.27 as reported similarly in a cerebellar tDCS experiment [17]. Therefore, twenty healthy females (age: 23.0±3.5years, height: 161.6±4.5 cm, body mass: 51.5±5.1 kg) with a regular menstrual cycle were recruited for the current study. All participants in this study had a right-sided dominant limb with uniform foot dominance. The leg’s length difference of participants was less than 1 cm and there was no any impact or other joint, musculoskeletal diseases on lower extremity that would affect their walking gait [8].

### Experimental design

This study employed a cross-over design and was single-blinded to participants (the investigators and corresponding authors of the project were aware of the randomization order and intervention allocation). In this study, the intervention of tDCS condition was provided by Halo Sport equipment, which is often a brain tDCS device manufactured by USA Halo Neuroscience. Three 24 cm^2^ primers with studded foam electrodes were soaked in saline solution before using to make electrical contact with the head more smoothly [2]. The associated mobile application with Halo Sport equipment were used to obtain microcurrent stimulation once started, the current reached about 2.0 mA within 30 s and lasted for 20 min at the same intensity to bilaterally stimulate the motor cortex of the brain [18]. The anodal electrode was located at Cz (Corresponding to the lower limbs area of primary motor cortex [19]), and the cathodal electrode was placed at approximately C5 and C6 [6] to stimulate the bilaterally motor cortex. The headphones played 20 min soothing music to divert participants’ attention both during tDCS and sham-tDCS conditions, however, the current was manually stopped of sham-tDCS condition only accompanied by music after the 30s current intervention. This study adhered to CONSORT guidelines for randomized controlled trials and had been registered on Chinese Clinical Trial Registry (ID: ChiCTR2100053942, on 04/12/2021).

### Experimental protocol

**Fig. 1:**
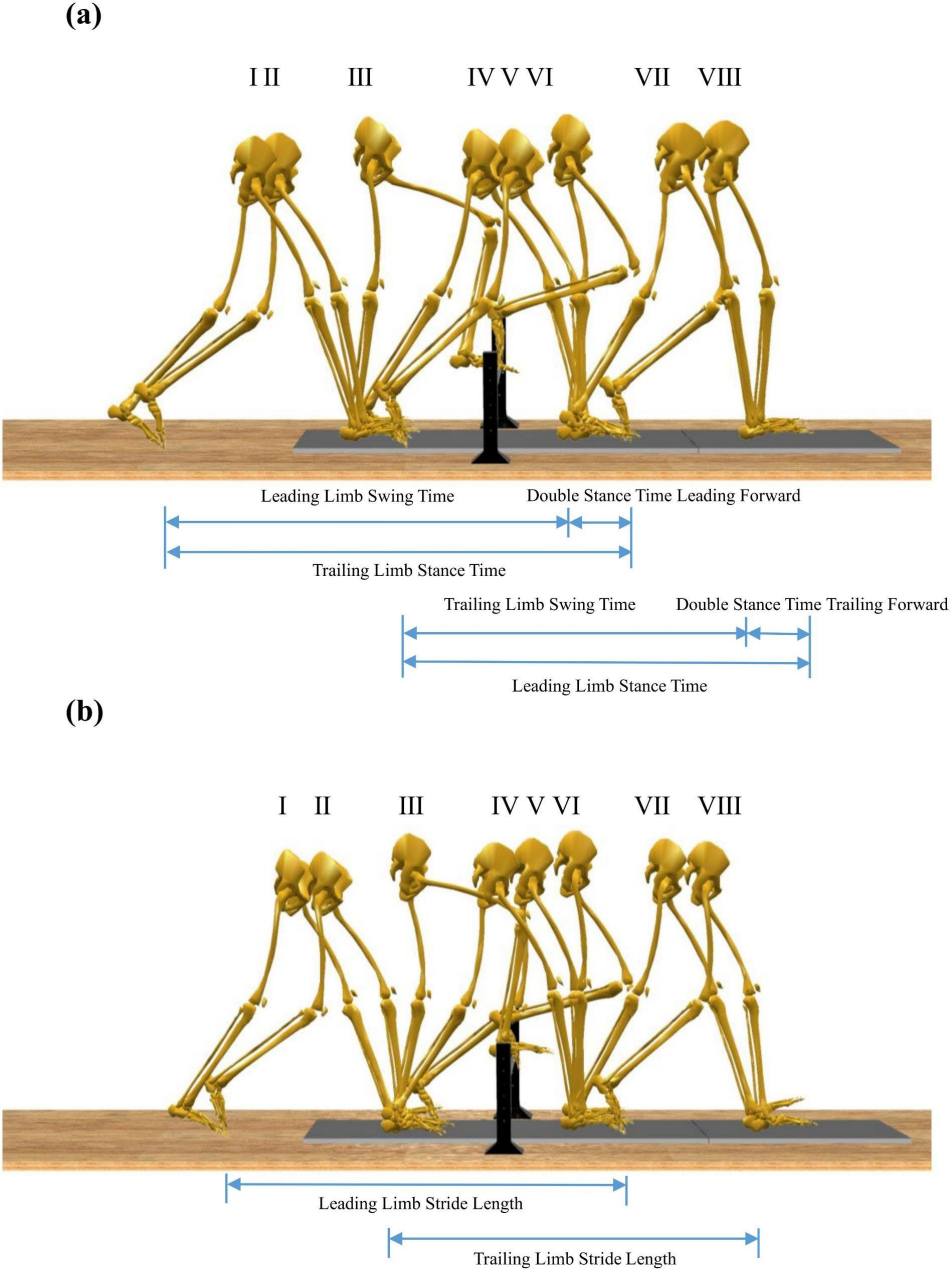
Gait cycle of crossing obstacles. (**a** & **b**: left and right limb, respectively, as leading limb during obstacle crossing; Eight instance: I, trailing limb heel strike event; II, leading limb toe-off event; III, leading limb toe-above obstacle event; IV, leading limb heel strike event; V, trailing limb toe-off event; VI, trailing limb toe-above obstacle event; VII, trailing limb heel strike event; VIII;, leading limb toe-off event)

The experiment consisted of two sessions and was separated by 5 days apart. During the first session, ten participants received tDCS stimulation, whereas the other ten participants with sham-tDCS intervention. In the second session, the sequence would be reversed. Accordingly, the tDCS or sham-tDCS stimulation order across the two sessions was counterbalanced. Plus, the order of crossing three different leg’s length obstacles height would be randomly assigned. All tests were completed within one month. The protocol of the obstacle crossing was illustrated in Fig. 1 where participants walked along the 8 m walkway. Prior to the formal experiment, each participant was subjected to at least twice simulation tests to familiarize themselves with the actual testing process. The subjects wore uniform clothing and shoes, and the data were collected six times for each height (each leg was collected three times as leading limb).

### Kinetic and kinematic data collection

Three-dimensional marker trajectory data were measured using a 10-camera motion analysis system (Vicon V5, Oxford Metrics Group, UK) at a sampling rate of 200 Hz. The ground reaction forces (GRF) were measured using three forceplates (AMTI BP600900, USA) at a sampling rate of 1200 Hz and two retro-reflective markers with 14 mm diameter were placed on the end of iron obstacle to define obstacle crossing gait cycle. The modified Plug-in Gait marker set with nineteen retro-reflective markers defined a lower extremity model of seven-segment rigid link. Marker trajectories were used low pass fourth-order Butterworth filter with 10 Hz cutoff frequency for each trial.

### Data analysis

Differences of spatiotemporal parameters (SP) including stride length, swing time (SW), stance time (ST), swing/stance time and double support (DS) time gait asymmetry (GA) were calculated using the formula [20]:

$$ GA=\left|100\left[\text{ln}\left({SP}_{leading}/{SP}_{trailing}\right)\right]\right|$$

Maximum joint angles of left/right leading limb were obtained from toe-off event to heel strike event (Fig. 1 II-IV) for each trail. Maximum joint angles of left/right trailing limb were obtained from toe-off event to heel strike event (Fig. 1 V-VII) for each trail.

### Statistical analysis

Descriptive values were obtained and reported as means and standard deviation (SD). Homogeneity of variance was examined by Levene’s test and the normality of the data was examined by the Kolmogorov-Smirnov Test. Sphericity was assessed and where the assumption of sphericity could not be assumed, adjustments to the degrees of freedom were made (ε > 0.75 = Huynh-Feldt; ε < 0.75 = Greenhouse-Geisser). All statistical tests were conducted using MATLAB (version R2020a; MathWorks, Inc., Natick, MA). Two-way repeated measures ANOVA was used to evaluate the effect of conditions (tDCS and sham-tDCS) and heights (30%, 20% and 10% leg’s length) on the spatiotemporal and maximum joint angle parameters for obstacle crossing. In cases where main or interaction effects occurred, post hoc pairwise analyses were performed, using paired samples t-tests with Bonferroni correction. The statistically significant level was set at *P* < 0.05 and effect size of Cohen’s d (ES: “small” around 0.2, “medium” about 0.5, “large” greater than 0.8 [21]) were computed to verify differences between conditions.

## Results

### Gait bilateral asymmetry decreased after tDCS during crossing higher obstacles

**Fig. 2:**
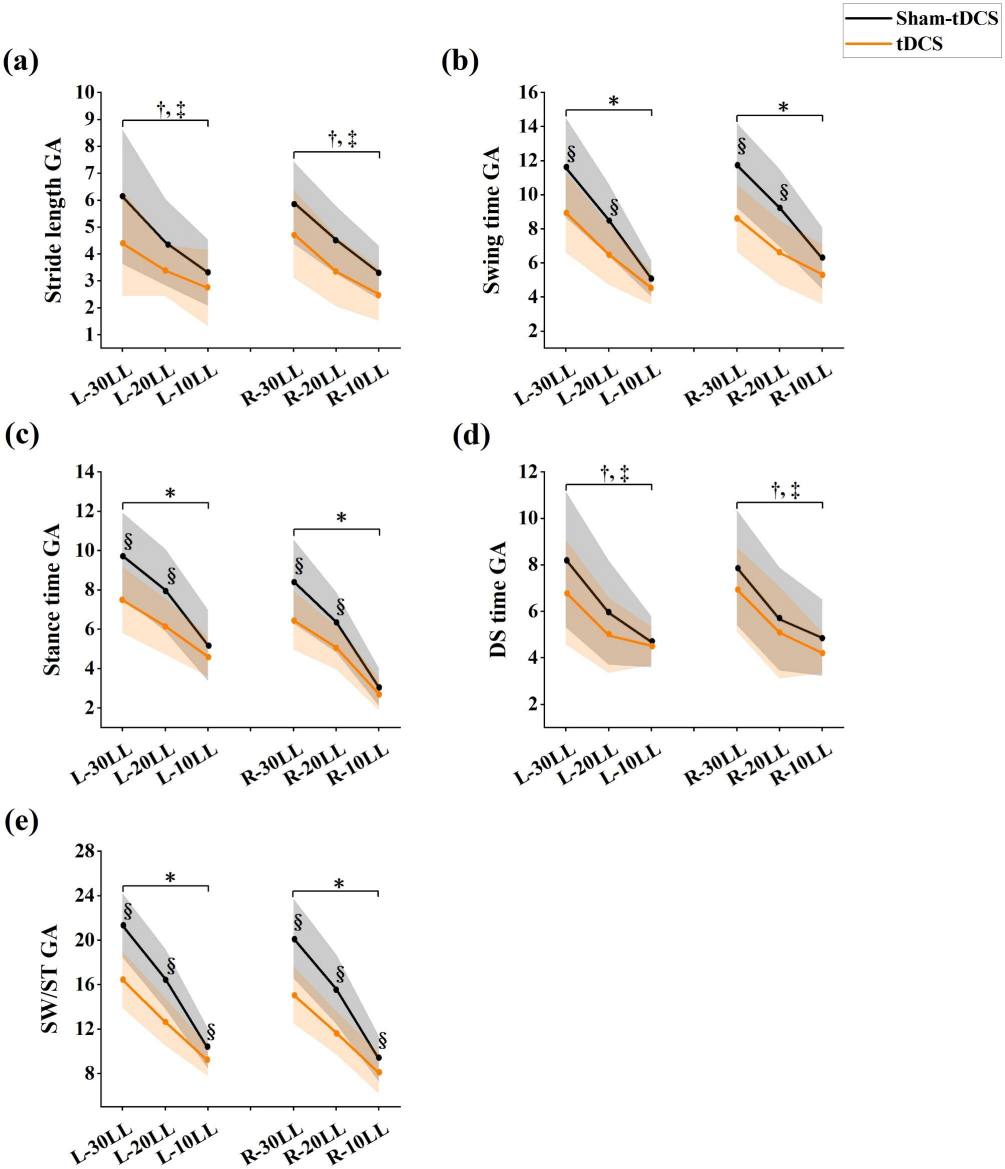
The parameters of gait asymmetry at tDCS condition and sham-tDCS condition among three obstacle crossing height. “*” indicates significant interaction effects; “†” indicates significant main effects between tDCS and sham-tDCS; “‡” indicates significant height main effects among different leg’s length; “§” indicates significant differences between tDCS and sham-tDCS treatment at each height. (L: Left/R: Right: left and right limb, respectively, as leading limb during obstacle crossing; 30, 20, 10LL: 30%, 20% and 10% leg’s length obstacles height; SW: swing time, ST: stance time, SW/ST: swing/stance time, DS: double support time, GA: gait asymmetry)

Gait bilateral asymmetry parameter values were shown in Fig. 2. There were no significant interaction between condition*height in left/right Stride length GA (Fig. 2a) (all, *P* > 0.305) and left/right DS time GA (Fig. 2d) (all, *P* > 0.278) between tDCS and sham-tDCS condition at 30%, 20%, 10% leg’s length obstacle height. However, left/right Swing time GA (*P* = 0.046, *P* = 0.038, respectively), left/right Stance time GA (all, *P* < 0.035), left/right SW/ST GA (all, *P* < 0.005) showed significant differences between condition and height interaction effects. Differences between sham-tDCS and tDCS conditions in Swing time GA (Fig. 2b), Stance time GA (Fig. 2c) and SW/ST GA (Fig. 2e) at three obstacle crossing heights were found (all *P* < 0.05). Compared with the sham-tDCS condition, the tDCS condition showed a significantly decreased in Swing time GA (Left: *P*_30%_=0.002, *P*_20%_=0.005, ES varying from 0.71 to 0.80; Right: *P*_30%_<0.001, *P*_20%_=0.002, ES varying from 0.78 to 1.04, respectively), Stance time GA (Left: *P*_30%_=0.001, *P*_20%_=0.003, ES varying from 0.76 to 0.83; Right: *P*_30%_=0.002, *P*_20%_=0.003, ES varying from 0.78 to 0.82, respectively) at 30% and 20% leg’s length obstacle heights but not with 10% leg’s length. Specifically, the SW/ST GA (Left: *P*_30%_<0.001, *P*_20%_<0.001, *P*_10%_=0.021, ES varying from 0.56 to 1.38; Right: *P*_30%_<0.001, *P*_20%_<0.001, p_10%_=0.045, ES varying from 0.48 to 1.40, respectively) of tDCS condition decreased significantly at 30%, 20% and 10% leg’s length obstacle height.

### Maximum joint angles decreased after tDCS during crossing higher obstacles

**Fig. 3:**
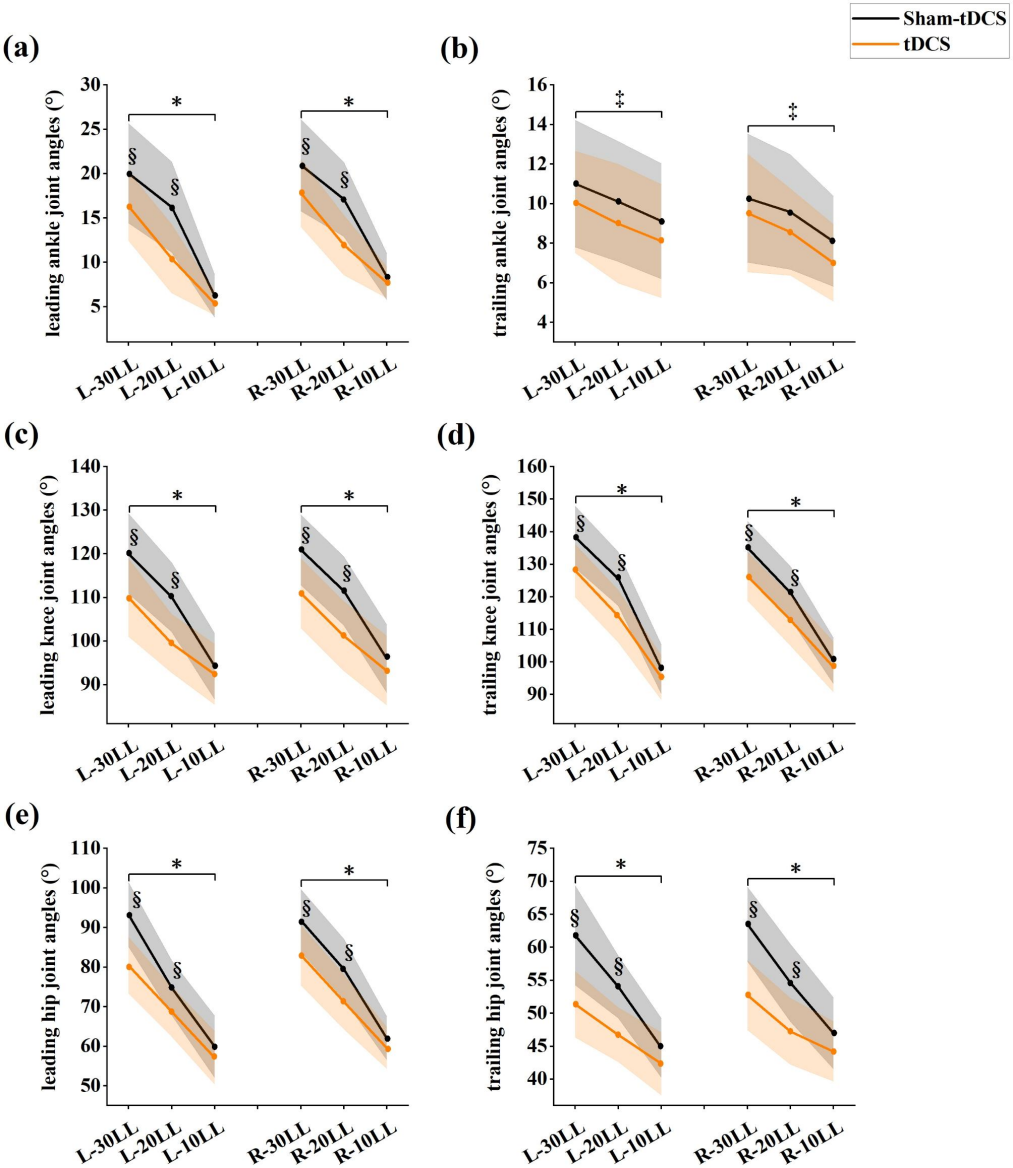
The parameters of leading/trailing limb maximum joint angles at tDCS condition and sham-tDCS condition among three obstacle crossing height. “*” indicates significant interaction effects; “†” indicates significant main effects between tDCS and sham-tDCS; “‡” indicates significant height main effects among different leg’s length; “§” indicates significant differences between tDCS and sham-tDCS treatment at each height. (L: Left/R: Right: left and right limb, respectively, as leading limb during obstacle crossing; 30, 20, 10LL: 30%, 20% and 10% leg’s length obstacles height)

Furthermore, leading and trailing limb maximum joint angles parameter values were shown in Fig. 3, an interaction effect was found between condition*height in left/right ankle (all, *P* < 0.025), left/right hip (all, *P* < 0.033); left/right knee (all, *P* < 0.048) of leading limb, left/right hip (all, *P* < 0.005), left/right knee (all, *P* < 0.029) of trailing limb, however, no significant interaction effect between condition*height was found for left/right ankle joint (Fig. 3b) when trailing limb crossed obstacles. Ankle joint angles (Fig. 3a), knee joint angles (Fig. 3c), hip joint angles (Fig. 3e) of leading limb and knee joint angles (Fig. 3d), hip joint angles (Fig. 3f) of trailing limb showed differences when compared sham-tDCS and tDCS condition. Specifically, ankle (Left: *P*_30%_=0.034, *P*_20%_=0.001, ES varying from 0.51 to 0.89; Right: *P*_30%_=0.036, *P*_20%_<0.001, ES varying from 0.50 to 1.01, respectively), knee (Left: *P*_30%_<0.007, *P*_20%_<0.001, ES varying from 0.67 to 1.18; Right: *P*_30%_=0.001, *P*_20%_=0.001, ES varying from 0.90 to 0.92, respectively), hip (Left: *P*_30%_<0.001, *P*_20%_=0.009, ES varying from 0.65 to 1.34; Right: *P*_30%_=0.001, *P*_20%_=0.002, ES varying from 0.79 to 0.89, respectively) joint angles of leading limb and knee (Left: *P*_30%_=0.001, *P*_20%_<0.001, ES varying from 0.84 to 1.03; Right: *P*_30%_<0.001, *P*_20%_<0.001, ES varying from 0.99 to 1.05, respectively), hip (Left: *P*_30%_<0.001, *P*_20%_<0.001, ES varying from 1.04 to 2.18; Right: *P*_30%_<0.001, *P*_20%_<0.001, ES varying from 1.27 to 1.49, respectively) joint angles of trailing limb in tDCS condition were lower than sham-tDCS condition at 30% and 20% leg’s length obstacle height but excluding 10% leg’s length obstacle height.

## Discussion

This is the first study to investigate the effect of tDCS in females when crossing the obstacle at various obstacle heights. This study has three major discoveries. First, at 30%, 20% leg’s length crossing height, the Swing time GA, Stance time GA, SW/ST GA decreased in the tDCS condition compared to the sham-tDCS. Second, leading limb hip-knee-ankle maximum joint angles decreased in the tDCS condition when compared with sham-tDCS. Lastly, trailing limb hip-knee maximum joint angles in tDCS condition were lower than sham-tDCS, suggesting that the tDCS intervention helped to reduce bilateral asymmetry when crossing the obstacle and changed joint angles. These results agree with our study hypothesis. Collectively, the finding of this study indicates that tDCS is able to enhance dynamic balance in healthy females when crossing obstacle at a more challenging task.

The results of this study found that Swing time GA, Stance time GA, and SW/ST GA were lower in the tDCS condition compared to the sham-tDCS condition at 30%, 20% leg’s length crossing height. The reciprocal inhibition between the hemispheres of healthy individuals via the transcallosal connections balances the motor excitability of both hemispheres [22]. Halakoo et al. revealed that the use of bilateral tDCS reduced the level of interhemispheric inhibition and superimposed bilateral hemispheric motor cortical excitability in healthy individuals [23]. In this study, the effect of bilateral tDCS may have a superimposed effect between the hemispheres to reduce the degree of neuromuscular drive between the dominant and non-dominant side of the lower limb. Consequently, the narrowing of differences led to a reduction in bilateral asymmetry at the tDCS condition when crossing an obstacle. This is evidenced by a reduction in Swing time GA, Stance time GA, and SW/ST GA. Moreover, as crossing height increases, the swing time of the leading limb lengthens [24] whilst the stable support of the trailing limb’s stance phase contributes to the safe crossing of the leading limb. Previous research has discovered that tDCS regulates the excitability of cortical areas involved in postural control [25]. In this current study, bilateral tDCS may activate corticospinal tract excitability and improve postural control of the non-dominant limb during the stance phase, thereby increasing non-dominant side support capacity and decreasing Stance time GA. Thus, the effect of tDCS microcurrent on spinal network excitability may apply to support postural stabilization and dynamic balance, making the Swing time GA, Stance time GA, and SW/ST GA of dominant versus non-dominant reduced at tDCS condition. However, there was no difference between the tDCS condition and the sham tDCS condition at 10% leg’s length crossing height, implying that crossing lower obstacle height was unaffected by the effect of tDCS to alter lower limb symmetry.

Compared to the sham-tDCS condition, the results of this study found that maximum hip-knee-ankle joint angles reduced of the left and right leading limb in the tDCS condition at 30%, 20% leg’s length crossing height. Previous research has found that the leading limb in young people increased the lower limb hip-knee-ankle flexion angle during obstacle crossing to accommodate the increased height of the obstacle such that the changes in joint kinematic patterns assist in reserving sufficient foot-obstacle clearance to complete a safe crossing [26]. Accordingly, for different obstacle heights, the end-point control of the limb is achieved by the joints movement to avoid contact with the obstacle and to reducfalling risk [26]. The ability of the body’s dynamic neural networks to innervate and control distal limb movements is critical for safe obstacle crossing. tDCS may increase the number of firing neurons by inducing excitability in intracortical and subcortical networks and enhancing the expression of synaptic plasticity [19, 27]. This subsequently enhances motor control in the lower limbs. Previous research has shown that anodal tDCS stimulation of the bilateral hemispheres’ primary motor cortex and premotor areas improved gait adaptation for lower limb movements, allowing the body to make correct motor decisions in response to situational changes [28]. In this study, tDCS stimulation on the motor cortex may have enhanced synaptic plasticity to increase the neural drive from the central nervous system to the leading limb during obstacle crossing, resulting in an increased end-point control of the lower limb to perform more precise crossing tasks. Thus, participants in the tDCS condition may be able to safely cross an obstacle with a smaller range of hip and knee flexion as well as ankle dorsiflexion angle of the leading limb for higher obstacle heights from an improvement of end-point control.

Compared to the sham-tDCS condition, the results of this study found that hip and knee maximum joint angles reduced of the left and right trailing limb in the tDCS condition at 30%, 20% leg’s length crossing height. Unlike the leading limb, the lack of visual cues increases the risk of falling when the trailing limb over an obstacle. Plus, the change in hip and knee angle plays a key role in trailing limb crossing. A past study found that bilateral anodal tDCS stimulation of the motor cortex for 20 min per day for 5 days in healthy subjects improved performance on alternating hand typing tasks [29]. In contrast, the present study was performed in a bilateral lower limb alternate crossing obstacle task immediately after a 20-minute bilateral anodal tDCS intervention. Connections within the M1 cortical region allow it to exhibit plasticity in response to external stimuli and thus alter the related nervous system-mediated motor functions [1]. Anodal tDCS stimulation of the M1 region induces specific changes in CM cells distributed on M1, which may enhance the motor function of target muscles and modulate human motor performance [30]. In this study, tDCS stimulation may enhance neuromuscular function and this could potentially alter the gait pattern during obstacles crossing. Moreover, the sustained effects of tDCS activation of the cerebral cortex premotor area can enhance lower limb motor function and agility [31]. tDCS technology for neuro-navigation of cortical functions is beneficial for gait optimization [32]. Thus, the intervention of the tDCS in this study enabled the trailing limb to make an accurate judgement easier to crossing higher obstacle height, certified by a smaller hip and knee angle for the trailing limb in the tDCS condition.

In summary, tDCS balanced the symmetry of spatio-temporal parameters between the dominant and non-dominant legs. At the same time, the tDCS intervention may improve the neuromuscular function and optimize the gait of the toe-off event to heel strike event in both the leading and trailing limb, resulting in safe crossing of the obstacle with a lower joint flexion angle. There are three limitations of this study. First, there may be a certain “ceiling effect” [4] on the functional neuroplasticity effect produced by tDCS in healthy people, namely, by inducing cortical plasticity while increasing voluntary activation to improve lower limb motor performance may only have an effect within a certain adaptive range. Secondly, the present study did not monitor the neural activity of the cerebral cortex after stimulation by tDCS and therefore could not examine the neurophysiological effects produced by tDCS during obstacle crossing at various leg’s length. Finally, the effect of the different menstrual phase on the crossing obstacles should be taken into account.

## Conclusions

We conclude that tDCS intervention is effective to enhance dynamic balance in female participants during obstacle crossing at the leg’s length above 20%, higher crossing heights require a high degree of coordination between the participants’ leading and trailing limbs. The reduced asymmetry of the spatio-temporal parameters and the altered lower limb joint angles can be derived from the fact that the tDCS technique contributes to postural control and dynamic stability when crossing an obstacle in healthy females. Based on the findings of this study, it may be possible to consider using bilateral anodal tDCS in healthy populations’ fitness activities to improve motor functionality and enable them to make accurate motor decisions in response to changes in their daily environment, the tDCS intervention has a potential preventive effect on safety issues that may arise when crossing obstacles.

### Electronic supplementary material

Below is the link to the electronic supplementary material.

Supplementary Material 1

Supplementary Material 2

## Data Availability

The data and materials used to support the findings of this study are available from the corresponding author upon request, and the datasets used and analyzed in the current study are included in this article.
