# Supplementary material for: The effect of transcranial direct current stimulation on bilateral asymmetry and joint angles of the lower limb for females when crossing obstacles
Source: BMC Sports Sci Med Rehabil. 2023 Dec 21;15:176. doi: 10.1186/s13102-023-00793-2 (PMC10734077; doi:10.1186/s13102-023-00793-2)
Supplement: Supplementary file 2 — Supplementary Material 2 [file 13102_2023_793_MOESM2_ESM.doc]

**Allocation**

**Analysis**

**Follow-Up**

**Enrollment**

Assessed for eligibility (n=20)

Excluded (n=0)

♦  Not meeting inclusion criteria (n=0)

♦  Declined to participate (n=0)

♦  Other reasons (n=0)

Analysed (n=20)
♦ Excluded from analysis (give reasons) (n=0)

Lost to follow-up (give reasons) (n=0)

Discontinued intervention (give reasons) (n=0)

tDCS intervention (n=20)

♦ Received allocated intervention (n=20)

♦ Did not receive allocated intervention (give reasons) (n=0)

Lost to follow-up (give reasons) (n=0)

Discontinued intervention (give reasons) (n=0)

Sham-tDCS intervention (n=20)

♦ Received allocated intervention (n=20)

♦ Did not receive allocated intervention (give reasons) (n=0)

Analysed (n=20)
♦ Excluded from analysis (give reasons) (n=0)

Randomized (n=20)

**Fig. S1 Consolidated Standards of Reporting Trials (CONSORT) flowchart of participants at each stage of the randomized trial**
